# Effects of Novel Preparation Technology on Flavor of Vegetable-Soy Sauce Compound Condiment

**DOI:** 10.3390/foods12061263

**Published:** 2023-03-16

**Authors:** Tiantian Tang, Min Zhang, Bhesh Bhandari

**Affiliations:** 1 State Key Laboratory of Food Science and Technology, Jiangnan University, Wuxi 214122, China; 2 International Joint Laboratory on Food Safety, Jiangnan University, Wuxi 214122, China; 3 Jiangsu Province International Joint Laboratory on Fresh Food Smart Processing and Quality Monitoring, Jiangnan University, Wuxi 214122, China; 4 School of Agriculture and Food Sciences, University of Queensland, Brisbane, QLD 4072, Australia

**Keywords:** vegetables, soy sauce, seafood, flavor, condiments

## Abstract

Vegetables contain important bioactive substances which have unique tastes and aromas and provide beneficial effects to human health. In this study, multiflavor blended soy sauce was prepared with the juice of eight kinds of vegetables, dried shrimp boiled stock, and six kinds of commercial soy sauce as raw materials, and thermal ultrasound was used as the sterilization method. The effects of adding different formulas of vegetable and seafood stock on the basic physical and chemical parameters, nutrition, antioxidant activity, flavor, and taste of soy sauce were investigated. The results showed that the basic physicochemical indices such as pH, total acid, color, soluble solids, and amino acid nitrogen of the product with a ratio of soy sauce to vegetable-seafood stock of 1:0.5 (*v*/*v*) could meet the production standards of soy sauce, and its flavor, taste, and sensory scores were relatively good, with the highest likeability (overall acceptability). The mixed soy sauce with a ratio of 1:2 (*v*/*v*) had higher vegetable and seafood flavors, and different vegetable flavors (celery, carrot, and onion) were more obvious, but its nutritional index was relatively low. Multiflavor vegetable-soy sauce can be used for quick cooking by chefs of catering enterprises, and may be used as a seasoning bag for prefabricated dishes and convenient foods, attracting increasing attention from manufacturers and consumers.

## 1. Introduction

Soy sauce is a condiment made from soybean, wheat, or corn and other grains using solid-state fermentation (koji) and brine fermentation (moromi). It has a strong umami taste and a very unique aroma [1] and is often used in Eastern and Western cuisines for dipping or stir frying, such as sushi, sashimi, fried noodles, salad, and various meat dishes. It can enhance the overall saltiness of various dishes, giving color and aroma to food, and is becoming more and more popular worldwide [2,3]. Flavor is a crucial attribute of soy sauce and is gradually becoming a key factor determining consumer acceptance and preference. It has been reported that nearly 300 different volatile compounds have been identified in soy sauce [4,5]. The formation of aroma compounds in traditional soy sauce is generally related to its key processing stages, including the cooking of raw materials, koji culture, moromi fermentation, and pasteurization.

In recent years, a variety of flavors of soy sauce have been developed, such as steamed fish soy sauce, seafood soy sauce, shiitake mushroom soy sauce, oak mushroom soy sauce, *Hericium erinaceus* fermented soy sauce, iron-fortified soy sauce, etc. It is worth noting that some vegetable soy sauce is attracting the attention of various condiment manufacturers and consumers. Although no relevant research articles have been reported so far, some patents have been published. Seong and Woong [6] proposed a kind of *Allium hookeri* soy sauce, in which the addition of *Allium hookeri* improved the storage performance and antioxidant effect of the soy sauce. Gong [7] reported a kind of fruit and vegetable health-care soy sauce. The preselected fruits and vegetables were washed, chopped, mixed with various spices, and boiled with water, followed by mixing with fermented soy sauce raw juice for thermal treatment to obtain the fruit and vegetable health soy sauce. Ok [8] added beef broth, fruit juice (brewed from apples and jujubes), and vegetable juice (brewed from garlic, kelp, onion, ginseng, and ginger) into soy sauce to produce a soy sauce mixture. Soo et al. [9] reported a unique-flavor soy sauce containing vegetables such as mustard, broccoli, cabbage, and Chinese cabbage. Ma [10] provided a processing method of *Toona sinensis* soy sauce. Ground *Toona sinensis* was added to soy to make a slurry, which was soaked in a closed container for more than 2 h, and finally, the soy sauce with a strong *Toona sinensis* flavor was filtered out. In addition, there are herb soy sauce [11], mustard soy sauce [12], *Rosmarinus officinalis* health-care soy sauce [13], and other soy sauce related products that have been patented. US condiment brand OhSaucy has launched a vegetable soy sauce, which can be used as an alternative to traditional soy sauce. Its main ingredients are brewing soy sauce, kelp concentrate, anchovy concentrate, fructo-oligosaccharides, mixed-vegetable concentrate, apple concentrate, pear concentrate, yeast extract, and water. On the basis of brewing soy sauce, seafood and the concentrated juice of vegetables and fruits are added, which not only reduces the salinity to the maximum, conforming to the healthy lifestyle of salt reduction, but also greatly improves the taste after cooking.

Soy sauce is an essential condiment for catering enterprises and chefs. Different soy sauces may have different tastes, and chefs may waste time using various soy sauces in the cooking process. In addition, the excessive use of soy sauce will make the color of the dishes dark brown or more salty, losing the original fragrance of the dishes. Therefore, according to the real demand of catering enterprises, this study aims to prepare a kind of multiflavor vegetable-soy sauce compound condiment suitable for fast cooking or convenient fast food. At the same time, the influence of adding vegetable juice on the nutritional ingredients and flavor of soy sauce is explored.

## 2. Materials and Methods

### 2.1. Materials and Reagents

Seafood soy sauce (Haitian), jinbiao soy sauce (Haitian), steamed fish soy sauce (Lee Kum Kee), spicy fresh dew (Knorr), fresh shellfish sauce (Totole), Maggi fresh (Nestle), celery, carrot, onion, green onion, red pepper, ginger, garlic, coriander, dried shrimps, dried shitake mushrooms, and rock sugar were purchased from local supermarkets in Wuxi, China. Food-grade ascorbic acid and potassium sorbate were purchased from Henan Wanbang Industrial Co., Ltd. (Zhengzhou, China). The juicer was purchased from Midea Group Co., Ltd. (Foshan, China).

Methanol (chromatographically pure) and acetonitrile (chromatographically pure) were purchased from Tedia Co., Ltd. (Fairfield, Ohio, USA). Tetrahydrofuran, triethylamine, hydrochloric acid, crystalline sodium acetate, and trichloroacetic acid were of analytical grade, and water was Millipore ultrapure water. Seventeen amino acid standards, including alanine (Ala), arginine (Arg), glycine (Gly), glutamic acid (Glu), aspartic acid (Asp), methionine (Met), serine (Ser), histidine (His), leucine (Leu), phenylalanine (Phe), isoleucine (Iso), cysteine (Cys), tyrosine (Tyr), lysine (Lys), threonine (Thr), valine (Val), and proline (Pro), were purchased from Sigma-Aldrich (Shanghai) Trading Co., Ltd. (Shanghai, China).

### 2.2. Preparation of Vegetable-Soy Sauce Compound Condiment

Seafood soy sauce, jinbiao soy sauce, steamed fish soy sauce, spicy fresh dew, fresh shellfish sauce, and Maggi fresh were mixed according to the volume ratio of 12:4:1:1:1:1 (*v*/*v*), and used as soy sauce mixed juice for later use. The celery, carrot, onion, green onion, red pepper, ginger, garlic, and coriander were mixed and squeezed according to the weight ratio of 15:1:5:1:1:1:1:1, 5:15:1:1:1:1:1:1, and 1:5:15:1:1:1:1:1 (*w*/*w*), respectively. After that, the mixed-vegetable juice was filtered through a 150-mesh sieve. A centrifuge (TG16-WS, Hunan Xiangyi Laboratory Instrument Development Co., Ltd., Changsha, China) was used to collect supernatant at 9000 rpm for 10 min. The three mixed-vegetable juices were, respectively, recorded as celery-flavored, carrot-flavored, and onion-flavored vegetable juices according to the highest added amount of vegetables. Amounts of 0.05% (*w*/*w*) ascorbic acid and 0.05% (*w*/*w*) potassium sorbate were added by weight of the vegetable juice. Dried shrimps (50 g), dried shiitake mushrooms (25 g), rock sugar (15 g), and distilled water (500 mL) were mixed and boiled for 10 min (about 200 mL of water remained). Above boiled juice was then diluted to 500 mL with distilled water. The juices were filtered through a 150-mesh sieve and centrifuged (9000 rpm, 15 min), and the supernatant was taken as seafood stock for later use. The vegetable juices with the three flavors were mixed with the seafood stock in a ratio of 1:1 (*v*/*v*) to obtain the mixed vegetable-seafood juice.

The soy sauce mixed juice and mixed vegetable-seafood liquid were mixed in a ratio of 1:0.5 and 1:2 (*v*/*v*), and centrifuged (9000 rpm, 10 min), and the supernatant was treated with thermal ultrasound at 100 kHz, 70 °C for 20 min. According to the ratio of soy sauce and 3 kinds of mixed vegetables and seafood stock (*v*/*v*), the final product was recorded as 1:0.5 (celery), 1:0.5 (carrot), 1:0.5 (onion), 1:2 (celery), 1:2 (carrot), and 1:2 (onion), and marked as CE1, CA1, ON1, CE2, CA2, and ON2, respectively. The samples mixed with soy sauce and distilled water at ratios of 1:0 (no addition), 1:0.5, and 1:2 (*v*/*v*) were used as blank controls and denoted as S, W1, and W2, respectively.

### 2.3. Analysis of Basic Physical and Chemical Parameters for Vegetable-Soy Sauce Compound Condiment

The colors of S, W1, CE1, CA1, ON1, W2, CE2, CA2, and ON2 soy sauce samples were determined using a colorimeter (CR-400, Konica Minolta Co., Osaka, Japan). Colors were represented by CIE a*, b*, and L* values, which represent redness/greenness, yellowness/blueness, and brightness, respectively. The pH of each soy sauce sample was determined using a pH meter (pHS-3C acidity meter; Shanghai Precision Science Instrument Co., Ltd., Shanghai, China). The total soluble solids (TSS) content was determined by a hand-held refractometer (Aipli Co., Hangzhou, China), expressed as °Brix. The total acid content of soy sauce was determined with alkaline titration. A 20 mL appropriately diluted sample was titrated to pH 8.2 with 0.1 N NaOH, and the result was reported as lactic acid content (g/100 mL). Salt content (NaCl, g/100 mL) was assessed by volumetric titration with AgNO_3_ using the Mohr’s method [14]. The salt content (dried at 105 °C to constant weight) was subtracted from the total solid content to give a non-salt soluble solid (g/100 mL). The amino acid nitrogen (AAN) in soy sauce was measured using the colorimetric method according to Chinese national standard GB 5009.235-2016. In sodium acetate-acetic acid buffer pH 4.8, amino acid nitrogen reacted with acetylacetone and formaldehyde to generate yellow 3,5-diacetic acid-2,6-dimethyl-1,4-dihydropyridine amino acid derivative. The absorbance was determined at a wavelength of 400 nm and quantified compared to the standard series.

### 2.4. Determination of Free Amino Acids

The determination of free amino acid content was slightly modified according to the method of Wu et al. [15] and Zheng et al. [16]. Sample pretreatment: 12.5 mL of 10% trichloroacetic acid (10 g/100 mL) was added to 1 mL of soy sauce (its weight was accurately recorded) and allowed to stand for 1 h. After that, the volume was fixed to 25 mL with distilled water. After mixing, the sample solution (1 mL) was transferred to a 1.5 mL centrifuge tube and centrifuged (15,000 rpm, 30 min). The supernatant was filtered with a 0.22 μm aqueous membrane and placed in a liquid-phase sample bottle.

HPLC analysis: Agilentl 100 HPLC system (Agilent Technologies Co. Ltd. (Palo Alto, CA, USA), including VWD detector (G1314A), autosampler (G1313A), quaternary pump (G1311A), and online degasser (G1322A). Mobile Phase A (pH 7.2): triethylamine, tetrahydrofuran, and 27.6 mmo/L sodium acetate (volume ratio 0.11:2.5:500). Mobile Phase B (pH 7.2): acetonitrile, methanol, and 80.9 mmol/L sodium acetate (volume ratio 2:2:1). Agilent Hypersil ODS column (5 μm, 4.0 mm × 250 mm). Gradient elution was performed with the following procedure: 0.0 min, 8% B; 17.0 min, 50% B; 20.1 min, 100% B; and 24.0 min, 0% B. The column temperature was 40 °C, and the mobile phase flow rate was 1.0 mL/min. The ultraviolet detector detection wavelength was 338 nm, and the proline detection wavelength was 262 nm. The external standard method was used for the quantification of amino acid content.

### 2.5. Determination of Total Phenolic and Flavonoid Content and Antioxidant Capacity

The total phenolic content of soy sauce was measured according to the Folin-Ciocalteu method reported by Li et al. [17]. Briefly, a 150-fold dilution of soy sauce (1 mL) was mixed with Folin phenol (1 mL) and reacted for 3 min, followed by the addition of 3 mL of sodium carbonate (75 g/L) and incubation in the dark for 90 min at room temperature. The absorbance was measured at 760 nm using a spectrophotometer (UV2600, TECHCOMP, China). The measurement results are based on the calibration curve of gallic acid, expressed as milligrams of gallic acid equivalent (GAE) per milliliter of soy sauce (mg GAE/mL). The total flavonoid was measured with the colorimetric method [18]. Briefly, 1 mL of soy sauce sample was added to 4 mL of distilled water and 0.3 mL of sodium nitrite solution (5%, *w*/*v*). After 5 min, 0.3 mL of aluminum chloride (10%, *w*/*v*) was added. After 6 min, 2 mL of NaOH (1 mol/L) was added and the volume was adjusted to 10 mL with distilled water. The absorbance was measured at 510 nm using a spectrophotometer. The results were calculated based on the standard curve of rutin, expressed as milligrams of rutin equivalent (RE) per milligram of soy sauce (mg RE/mL).

The DPPH radical scavenging activity of soy sauce was determined using the method reported by Shi et al. [19]. The diluted soy sauce sample was mixed with the DPPH solution and reacted at room temperature in the dark for 30 min, and the absorbance measurement wavelength was 517 nm. The scavenging activity of ABTS radicals was determined according to the method described by Qiu et al. [20]. ABTS radical reserve solution was prepared by mixing ABTS aqueous solution with potassium persulfate solution in equal amounts and leaving it to stand in the dark at room temperature for 12 h. The diluted soy sauce sample was mixed with ABTS radical reserve solution, and then reacted in the dark at room temperature for 15 min, and the absorbance was measured at a wavelength of 734 nm.

### 2.6. Electronic Nose Analysis

The flavor of each soy sauce product was analyzed with an electronic nose (iNose, Isenso, Ruifen Trading Co., Shanghai, China). Table 1 shows the responsive compounds corresponding to the 18 sensor array systems of the electronic nose. Soy sauce (4 mL) was placed in a glass bottle specifically designed for electronic nose testing and allowed to stand for 2 h under a seal to enrich the sufficient flavor compounds, following the method of Zhang et al. [21] with slight modifications. The sensor was cleaned and calibrated with an air flow rate of 1 L·min^−1^ for more than 50 min before the start of the experiment. Each sample had the same measurement time of 60 s, sensor wash time of 120 s, and air flow rate of 1 L min^−1^. At the end of the experiment, the sensor was cleaned for more than 30 min.

### 2.7. Electronic Tongue Analysis

According to the description of Zheng et al. [23] and Lao et al. [24], the taste of soy sauce was tested using a commercial electronic tongue instrument (Insent, taste sensing system SA402B, Intelligent Sensor Technology, Inc., Kanagawa, Japan). The electronic tongue was capable of measuring eight basic sensory qualities including sourness, saltiness, bitterness, bitter aftertaste (aftertaste-B), astringency, astringent aftertaste (aftertaste-A), richness, and umami. The taste reference solution was configured to contain 2.2365 g of KCl and 0.045 g of tartaric acid in 1000 mL of distilled water, and the sensor response of this reference solution was zero. The soy sauce was diluted 30 times to meet the requirements of determination. The measurements were started when the biofilm sensor (taste sensor) was stable, and after a single measurement, the sensor was cleaned to measure the next sample.

### 2.8. Sensory Evaluation

Sensory evaluation was conducted within 5 h after soy sauce production in the Research Center for Food Resources and Comprehensive Utilization of Jiangnan University. The sensory evaluation group consisted of 20 well-trained individuals in the 20–40 age group from Jiangnan University. All panel members were asked to avoid stimulating food, alcohol, and tobacco use for 8 h prior to the assessment and rinsed their mouth to clean their sense of taste before each sample was evaluated. The sensory analysis of color, appearance, soy sauce flavor, vegetable flavor, seafood flavor, and likeability (overall acceptability) of S, W1, CE1, CA1, ON1, W2, CE2, CA2, and ON2 soy sauces was performed by these team members. According to Table 2, the score for each evaluated attribute ranged from 1 (very disliked) to 9 (very liked).

### 2.9. Statistical Analysis

Electronic nose and electronic tongue experiments were repeated 5 times. Other experiments were repeated 3–6 times. GraphPad Prism 9.0 (GraphPad Software Inc., San Diego, CA, USA), Origin 2018 (OriginLab, Northampton, MA, USA), and SPSS 26.0 (IBM, Chicago, IL, USA) were used for plotting and data analysis. At the 95% confidence level, significant differences between the means were determined using a one-way analysis of variance (ANOVA). Different letters were used to indicate the significant difference of data (*p* < 0.05), and experimental result data were expressed as mean ± SD.

## 3. Results and Discussion

### 3.1. General Physicochemical Property Analysis

Table 3 shows the results of determination of general physicochemical properties of vegetable juice-soy sauce compound condiment. The color directly affects the appearance quality of soy sauce and consumers’ purchase intention [25]. The red/dark brown color of soy sauce is the result of the Maillard reaction of reducing sugars and amino acids produced by the enzymatic hydrolysis of carbohydrates and proteins during fermentation [26]. According to Table 3, the difference in brightness (L*) of the soy sauce with different formulations was not significant. Compared to W1 and W2, the soy sauce with the addition of vegetable-seafood stock had a higher redness value (a*) and yellowness value (b*), which might be due to the yellowish color of carrots and boiled shrimp skin. In addition, compared to pure soy sauce S, the overall color of the compound soy sauce product became lighter due to the dilution effect. It was previously reported that either too-low or too-high L* values did not affect soy sauce appearance, while high a* and b* values were important contributors to high-quality soy sauce [27]. Light-colored soy sauce was generally more popular than traditional soy sauce [28]. In the development of new soy sauce products, controlling the color of soy sauce is beneficial to improving customer acceptance.

The pH plays a vital role in the process of moromi fermentation. Microbial fermentation, substance hydrolysis, microbial cell autolysis, and the release of organic acids, free fatty acids, and amino acids can all lead to decreases in pH [29]. Typically, Japanese soy sauce has a pH between 4.6 and 4.9 [27,28]. Syifaa et al. [30] measured a pH range of 4.01–4.88 for common soy sauces in Southeast Asia. Commercially available soy sauce in Brazil had a pH between 4.00 and 5.27 [31]. The pH of the radish-, apple-, and pear-fermented soy sauce products ranged from 4.85 to 5.47 [32]. In this study, the pH values of all soy sauce products were in the range of 5.10–5.24; the pH value of pure soy sauce S was the lowest at 5.10. The larger the ratio of vegetable to seafood juice was, the higher the pH value was. The total acid content of soy sauce increased continuously during the fermentation process due to the production of organic acids. The highest total acid content of pure soy sauce S was 1.51 g/100 mL (calculated based on lactic acid), and with the addition of vegetable-seafood juice, the total acid content decreased due to the dilution effect. The 1:2 formula soy sauce samples (W2, CE2, CA2, and ON2) had the lowest total acid content, and there was no significant difference between the samples. Hoang et al. [33] recommended that the total acid content in soy sauce products should not exceed 2.0 g/100 mL and be less than 1.4 g/100 mL. The Chinese Soy Sauce Hygiene Standard GB 2717-2003 stipulated that the total acid content in soy sauce should be less than 2.5 g/100 mL (calculated based on lactic acid).

The TSS content of pure soy sauce S was the highest (39.50 °Brix), and the 1:0.5 soy sauce (27.88–29.75 °Brix) contained more soluble solids than the 1:2 soy sauce (17.75–20.75 °Brix). Compared to W1 and W2, the content of TSS increased through the addition of vegetable and seafood juice. In general, soy sauce has a higher concentration of sodium chloride to prevent microbial spoilage. The sodium chloride and non-salt-soluble solid contents of pure soy sauce S were up to 18.33 g/100 mL and 19.17 g/100 mL, respectively. The sodium chloride and non-salt-soluble solid contents of 1:0.5 soy sauce juice ranged from 12.87 to 13.65 and 11.43 to 12.64 g/100 mL, and that of 1:2 ranged from 8.78 to 8.97 and 3.08 to 6.65 g/100 mL. The soy sauce with added vegetable-seafood juice had a higher sodium chloride content than W1 and W2, which might be due to the salt in the dried shrimp being boiled in the seafood stock. Previous studies have shown that the TSS content of soy sauce is generally in the range of 24.8–57.9 g/100 mL, and the salt content is in the range of 11.09–22.18 g/100 mL [34]. Li et al. [35] measured soy sauce, and it contained 38.6 g/100 mL TSS and 18.9 g/100 mL non-salt soluble solids. Amino acid nitrogen content is considered as the main parameter to evaluate the quality of soy sauce products [36]. According to the current Chinese standard, the amino acid nitrogen content of fermented soy sauce should be higher than 0.4 g/100 mL (GB 2717-2018). The amino acid nitrogen content in that blended soy sauce was generally less than 0.4 g/100 mL [37]. The amino acid nitrogen content of pure soy sauce S was 0.79 g/100 mL, and that of W1, CE1, CA1, and ON1 were 0.49, 0.59, 0.56, and 0.54 g/100 mL, respectively. However, the amino acid nitrogen content of 1:2 soy sauce products was lower than 0.4 g/100 mL. In summary, most of the physicochemical parameters of pure soy sauce decreased after the addition of vegetable juice due to the dilution effect, and the soy sauce juice with the formula of 1:0.5 basically met the general quality standards for the production of soy sauce.

### 3.2. Free Amino Acids (FAAs)

During the fermentation of soy sauce, the action of protease and glutaminase leads to the hydrolysis of proteins and the formation of FAAs [38]. Free amino acids are considered important contributors to the unique taste of soy sauce. A quantitative analysis of 17 common FAAs in blended soy sauce samples was conducted in this study (Table 4). It was found that Glu, Asp, Leu, Pro, and Val were the main amino acids in soy sauce, especially glutamic acid, which accounted for more than 50% of the total FAA content and was considered to be an important contributor to the flavor of soy sauce. The umami amino acids represented by Glu and Asp accounted for about 60% of the total FAA content. Glu is an essential umami substance, and other amino acids can promote umami through synergistic effects [28]. The proportion of sweet (16.73–18.71%) and bitter (20.46–22.64%) amino acids in the total FAA content was not significantly different among different soy sauce samples and was generally lower than the umami (59.12–61.97%) amino acid content. These results were basically consistent with the report by Kong et al. [39], where the content of umami amino acids in Chinese commercial soy sauce accounted for 28.83–97.18% of the total FAAs, with an average of 62.42%, and Glu, Leu, Ala, and Pro were the main free amino acids. By contrast, the soy sauce used in this research was mainly seafood soy sauce with high umami, and the proportion of umami amino acids was significantly higher than that of soy-brewed soy sauce. It was reported that the umami amino acids (Glu and Asp) in soy sauce made from soybean accounted for more than 23.94% of the total FAA content [38]. Similarly, Lin et al. [40] measured the umami amino acids of soy sauce made from soybeans, finding that they accounted for 6.06–25.20% of the total FAAs.

As expected, S had the highest content of FAAs (7.86 g/100 mL), followed by CE1 (6.26 g/100 mL), CA1 (6.10 g/100 mL), W1 (5.62 g/100 mL), and ON1 (5.44 g/100 mL), and the 1:2 soy sauce formulation generally had low FAA content (about 3.00 g/100 mL). The total FAA content of CE1 and CA1 was 11.39% and 8.54% higher than that of W1, respectively, and the Glu content was 11.61% and 9.68% higher than that of W1, respectively. This may be due to the addition of seafood juice containing a certain amount of free amino acids. Glu, Gly, and Ala are considered to be the main flavor amino acids in oyster juice [41]. The most abundant amino acids in shrimp cooking juice are, in sequence, Glu, Gly, Pro, Asp, and Arg [42]. Gly, Ala, Glu, and Arg account for about 40% of the total FAAs in oyster cooker effluent [43]. In addition, vegetable juice also contains a certain amount of free FAAs. For example, the total FAA concentration of broccoli juice is 256.66 mg/100 mL, and Arg accounts for 21% [44]. Therefore, adding vegetable-seafood juice is helpful to increase the content of FAAs in soy sauce, but at a ratio of 1:0.5, it will not exceed the content of FAAs in pure soy sauce due to the dilution effect.

### 3.3. Total Phenolic, Flavonoid and Antioxidant Activity

**Figure 1:**
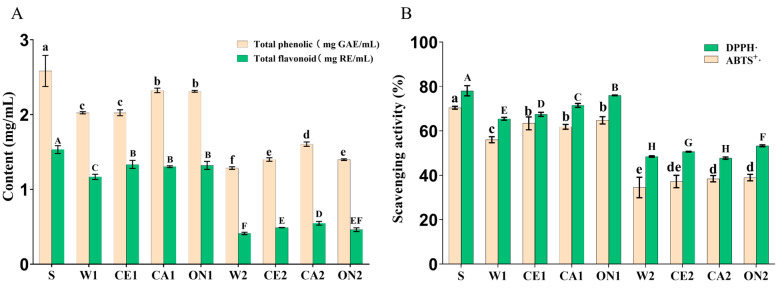
Total phenolics, flavonoids (**A**), and antioxidant capacity (**B**) of soy sauce. Different letters represent significant differences between groups (*p* < 0.05).

The total phenolic and total flavonoid contents of pure soy sauce S reached 2.58 mg GAE/mL and 1.53 mg RE/mL, respectively (Figure 1A). The total phenolic content was similar to the result previously reported by Gao et al. [45] and higher than that of citrus-peel-fermented soy sauce [46]. Phenols and flavonoids are secondary metabolites abundantly present in fruits and vegetables, which significantly promote human health. The total phenolic and flavonoid contents of the soy sauce with the formula of 1:0.5 and 1:2 with added vegetable juice were higher than those of the control group (W1 and W2) and lower than S. The total phenolic contents of CA1 and ON1 were 14.85% and 14.36% higher than that of W1, respectively, and the flavonoid contents of CE1, CA1, and ON1 were 13.68%, 11.11%, and 12.82% higher than that of W1, respectively. Previous studies have shown a close positive correlation between the content of phenolic compounds and antioxidant activity [47,48].

Figure 1B shows the antioxidant activity of the soy sauce products. S had the highest antioxidant activity, with the scavenging abilities of ABTS and DPPH radicals of 70.40% and 78.04%, respectively. Basically consistent with the change trend in total phenolic and flavonoid content, the free radical scavenging ability of the 1:0.5 and 1:2 formula soy sauce with added vegetable juice was higher than that of the control group (W1 and W2) and lower than that of S. The total phenolic contents of CE1, CA1, and ON1 were 13.22%, 10.38%, and 15.63% higher than those of W1, respectively, and the flavonoid contents were 13.68%, 11.11%, and 12.82% higher than those of W1, respectively. CE1, CA1, and ON1 had better antioxidant activity because they contained celery, carrots, and onions, etc., and their potential role as an antioxidant has been well documented in the literature [49,50]. In addition, many reports indicated that amino acids have strong antioxidant activity [45,51], which may be one reason for the better antioxidant properties of vegetable-seafood soy sauce. Fan et al. [52] reported that aromatic amino acids and histidine are considered effective radical scavengers because they can easily provide protons to electron-deficient radicals while maintaining their stability through resonance structures. Amino acids can act as a synergistic antioxidant as a natural component of food materials [53].

### 3.4. Electronic Nose Analysis

**Figure 2:**
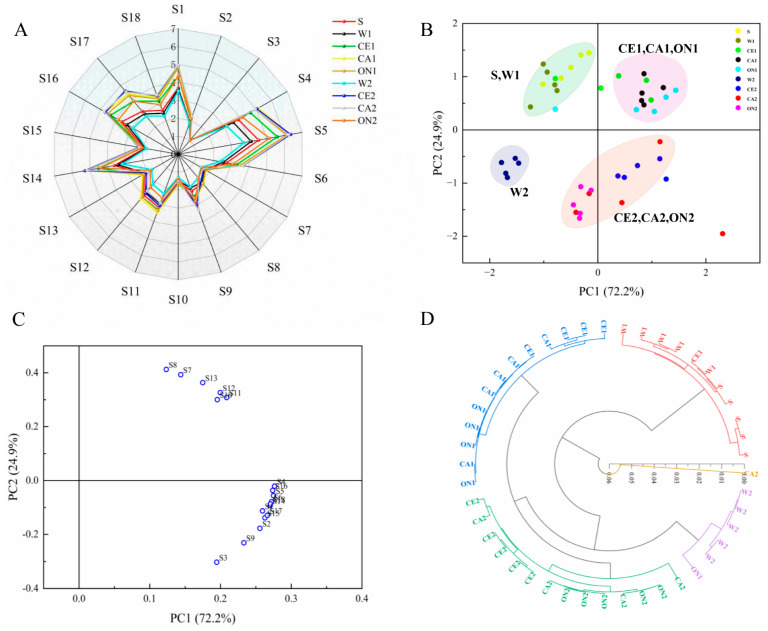
Radar plot (**A**), PCA score plot (**B**), loading plot (**C**), and HCA analysis plot (**D**) of soy sauce based on e-nose response data.

Electronic noses are sensitive to the overall smell of food, and slight changes in the presence of volatile compounds can cause differences in the sensor response [54]. Electronic noses are a common tool for analyzing the flavor changes in soy sauce [55,56]. Figure 2A shows the radar map of the odor distribution of the soy sauce with different formulations. The sensors with relatively strong responses to soy sauce samples were S1, S4, S5, S6, S9, S11, S12, S14, S16, S17, and S18, which were, respectively, sensitive to alkanes, sulfides, organic amines, aromatic compounds, aldehydes, alkenes, short-chain alkanes, flammable gases, sulfides, nitrides, and alcohols. The sensor response values of the S, W1, and W2 groups decreased successively, indicating that the overall flavor of the soy sauce became weaker with the increase in dilution degree. The sensor response value of soy sauce with added vegetable-seafood juice was larger, and the soy sauce flavor response value of the 1:2 formula was higher than that of the 1:0.5 formula, which indicated that the addition of vegetable-seafood juice enhanced the overall flavor of the soy sauce.

A principal component analysis (PCA) was performed on the response signals of the 18 sensors for each sample (Figure 2B). The contribution rates of the first and second principal components were 72.2% and 24.9%, respectively, and the total contribution rate was 97.1%, which covered almost all the variable information, indicating that the principal components could reflect all the characteristics of the volatile odor of different soy sauce samples. CE1, CA1, and ON1 samples were mainly distributed in the first quadrant with relatively close distances, and the differences between the samples were small. CE2, CA2, and ON2 were mainly distributed in the third and fourth quadrants, with little difference among the samples. S and W1 were in the second quadrant, while W2 was far away from other samples. This indicated that the samples of the soy sauce group (S, W1, and W2), the 1:0.5 soy sauce, and the 1:2 soy sauce were distributed in their independent regions, which were clearly distinguished by the PCA, and the flavor differences among the three groups were larger. However, the difference in flavor was small among different vegetables (celery, carrot, and onion). In the loading diagram (Figure 2C), the coordinates of each sensor can accurately reflect its contribution to the volatile odor of the sample. The farther from the origin, the greater the sensor’s contribution to the principal component, and vice versa. Among the eighteen sensors, the sensors that contributed more to the first principal component were S4, S16, S5, and S6, and the one that contributed the most to the second principal component was S8. This indicated that sulfide, organic amine, aromatic compounds, and short-chain alkane played the main roles in the differentiation of soy sauce with different formulations.

A hierarchical cluster analysis (HCA) was performed on the response values of 18 sensors to visually and objectively clarify the flavor differences between the samples. The earlier the samples were gathered into the same group, the closer they became. As shown in Figure 2D, the soy sauce samples were mainly classified into four groups. S and W1 were clustered into one group; W2 was clustered into one group individually; CE1, CA1, and ON1 were clustered into one group; and CE2, CA2, and ON2 were clustered into one group. The HCA results were consistent with the PCA results. Compared to the control W1, the addition of vegetable-seafood juice could change the flavor of soy sauce and significantly improve the aroma quality, but the flavor differences between the celery, carrot, and onion soy sauce samples were small.

### 3.5. Electronic Tongue Analysis

**Figure 3:**
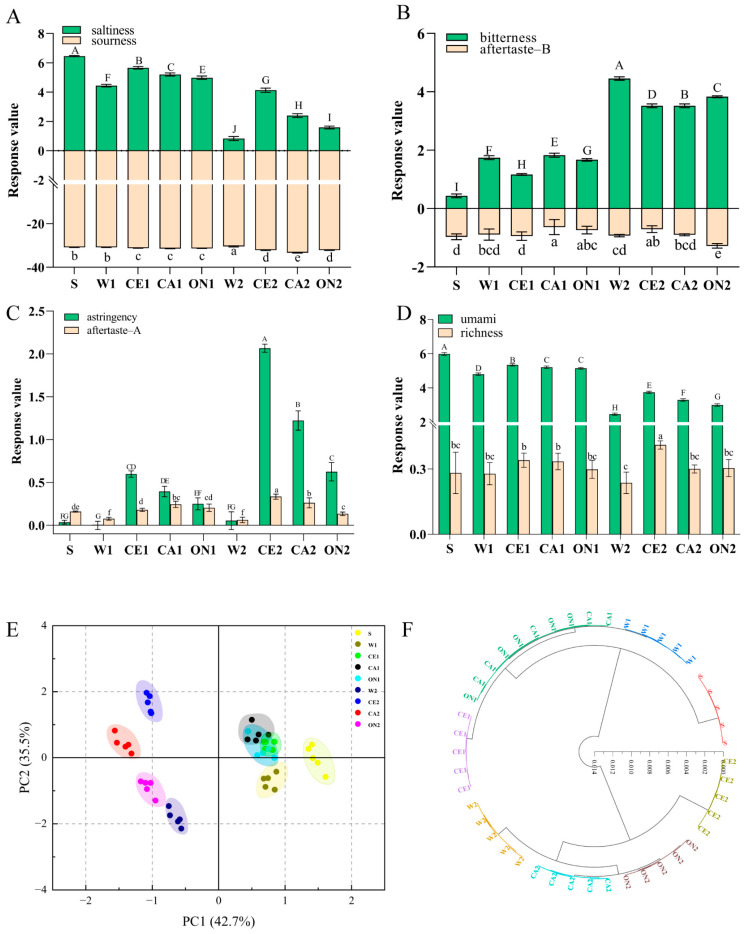
Saltiness and sourness (**A**), bitterness and bitterness aftertaste (**B**), astringency and astringency aftertaste (**C**), umami and richness (**D**) of different soy sauces; PCA score plot (**E**) and HCA analysis plot (**F**) of soy sauce taste based on electronic tongue response data. Different letters represent significant differences between groups (*p* < 0.05).

The electronic tongue uses artificial lipid membrane sensor technology to quantitatively analyze the six basic tastes of soy sauce: sourness, saltiness, astringency, aftertaste-A (astringency aftertaste), bitterness, aftertaste-B (bitterness aftertaste), richness, and umami [57]. Before determining the taste of the soy sauce, the taste of the distilled water used for preparing the soy sauce was first measured. The results showed that the saltiness, sourness, bitterness, aftertaste-B, astringency, aftertaste-A, umami, and richness of the distilled water were −20.11, −17.83, 16.13, −0.73, −4.43, −0.39, −5.30, and 0.35, respectively. Figure 3A shows the saltiness and sourness of soy sauce. Pure soy sauce S had the most salty taste, followed by the soy sauce with a formula of 1:0.5. The salty taste of CE1, CA1, and ON1 was higher than that of W1, which might be due to the salty taste of boiled seafood sauce. The sourness of the sauce was caused by lactic acid and other organic acids produced by lactic acid bacteria fermentation [58]. The sourness of the soy sauce did not change significantly after adding vegetable-seafood juice, and the sourness of all the samples was lower than that of the distilled water. The bitterness and umami of soy sauce are related to the amino acids produced during the fermentation process. After the soy sauce was diluted (W1 and W2), its bitterness significantly increased (Figure 3B), mainly due to the presence of a certain bitterness in distilled water [59]. The bitter aftertaste of all the soy sauce samples was lower than that of the distilled water. The variation trend in the astringency and astringency aftertaste of the soy sauce was basically the same. That is, the astringency and astringency aftertaste of the soy sauce increased significantly after adding vegetable and seafood juice, and the order was celery > carrot > onion-flavored soy sauce (Figure 3C). The umami taste of the soy sauce decreased after dilution. Compared to the control groups W1 and W2, the umami of the vegetable-seafood soy sauce was significantly increased, but still lower than that of the pure soy sauce (Figure 3D). The richness of CE2 was the highest, which may be due to the obvious taste of celery.

Figure 3E shows the PCA scores of different soy sauce seasonings based on the electronic tongue response data. PC1 and PC2 accounted for 42.7% and 35.5% of the total variance, respectively, for a total contribution of 78.2%. The scores showed that the soy sauce groups (S, W1, and W2) could be distinguished to a certain extent. CE1, CA1, and ON1 were close to each other, indicating that their tastes were similar. Soy sauce samples that were 1:2 formulated (W2, CE2, CA2, and ON2) showed good overall discrimination and large taste difference. HCA was performed on the response data of the electronic tongue. As shown in Figure 3F, the soy sauce condiments with different formulations were mainly divided into eight categories. S, W1, CE1, W2, CE2, CA2, and ON2 could be clustered into one group alone, indicating that there were significant differences in taste among different samples. CA1 and ON1 were gathered into one group, indicating that the tastes were similar. This result was basically consistent with that of the PCA. The taste of the soy sauce after adding vegetable-seafood juice was different from that of the pure soy sauce or diluted soy sauce, and there were some differences in the taste of the soy sauce from different vegetables (celery, carrot, and onion).

### 3.6. Sensory Evaluation

**Figure 4:**
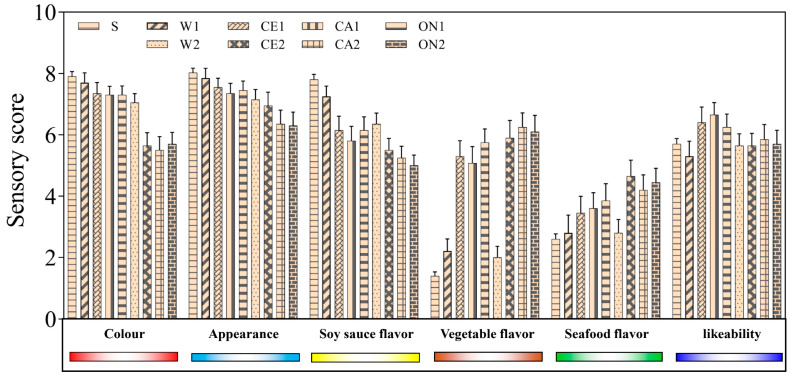
Sensory evaluation results of soy sauce with different formulations.

Twenty panel members conducted a sensory evaluation on the color, appearance, soy sauce flavor, vegetable flavor, seafood flavor, and likeability (overall acceptability) of the soy sauce products. As shown in Figure 4, S and W1 had the highest color and appearance scores, followed by the 1:0.5 vegetable soy sauce (CE1, CA1, and ON1), with both color and appearance scores greater than 7. This indicated that the color and appearance of soy sauce would decrease with the addition of vegetable-seafood juice, and the 1:0.5 formula had little influence on the soy sauce. For the flavor of the soy sauce, S, W1, and W2 scored the highest, followed by the 1:0.5 vegetable formula soy sauce. CE2, CA2, and ON2 had the highest scores for vegetable and seafood flavor, while S, W1, and W2 had the lowest scores. This showed that the more vegetable and seafood juice added, the lower the flavor of soy sauce and the higher the flavor of vegetables and seafood would be, and this flavor difference could be identified by human senses. In terms of the popularity of soy sauce products, the 1:0.5 vegetable-seafood soy sauce (CE1, CA1, and ON1) had the highest score, indicating that consumers might have a high acceptance rate of mixed-vegetable soy sauce.

## 4. Conclusions

In this study, according to the catering enterprises and the real demand, a variety of flavors of mixed-vegetable seafood soy sauce were developed, and the influence of different formula proportions on the physical and chemical parameters, nutrition, flavor, and taste of soy sauce was studied. The results showed that the product with the ratio of soy sauce to vegetable-seafood juice of 1:0.5 (*v*/*v*) had relatively good flavor, mouthfeel, and sensory scores, but the flavors of different vegetables (celery, carrot, and onion) were difficult to distinguish. It is suggested that manufacturers or catering enterprises should control the ratio of vegetables to soy sauce within 1:0.5 and strive to develop more soy sauce with multiple flavors to meet the needs of consumers or chefs.

## Figures and Tables

**Table 1:** Response substances and substance categories corresponding to the sensor [22].

Sensor	Response Substance	Substance Category
S1	Alkanes and smog	Propane, natural gas, and smog
S2	Alcohols, aldehydes, and short-chain alkanes	Alcohol, smog, isobutane, and formaldehyde
S3	Ozone	——
S4	Sulfides	Hydrogen sulfide
S5	Organic amines	Ammonia, methylamine, and ethanolamine
S6	Organic gases, phenylketones, aldehydes, and aromatic compounds	Toluene, acetone, ethanol, hydrogen, and other organic vapors
S7	Short-chain alkanes	Methane, natural gas, and methane
S8	Short-chain alkanes	Propane and liquefied gas
S9	Aromatic compounds and aldehydes	Toluene, formaldehyde, benzene, alcohol, and acetone
S10	Hydrogen-containing gases	Hydrogen
S11	Alkanes and olefin	Liquefied gas, alkane, and alkene
S12	Short-chain alkanes	Liquefied gas and methane
S13	Combustible gases	Methane
S14	Flammable gas	Flammable gas and smoke
S15	Alkane and organic gas	Smoke, isobutane, organic acid esters, and aliphatic hydrocarbons
S16	Sulfides	Sulfur compounds
S17	Nitrides	Nitrogen oxides
S18	Ketone and alcohol	Acetone, ethanol, and organic solvent

**Table 2:** Sensory scoring parameters.

Attribute	Descriptive Terms	Full Score of 9 (Minimum of 1 is Extremely Poor or Disliked)
Colour	Thick, black, reddish brown or tawny, shiny, and bright.	Glossy and bright color (6–9). Incorrect color (1–5).
Appearance	Concentration, viscosity, clarity, the presence of precipitation, and the presence of suspended solids.	Thin and moderately thick, clear and transparent, no precipitation, no suspended matter, and uniform liquid (6–9). Thin and not moderate, with suspended sediment (1–5).
Soy sauce flavor	Rich sauce flavor, mellow flavor, ester flavor, koji flavor, caramel flavor, amino acid flavor, ammonia gas, and musty flavor.	Soy sauce with strong flavor such as sauce flavor, mellow flavor, and ester flavor (6–9). Soy sauce has low flavor, burnt smell, musty smell, and ammonia gas (1–5).
Vegetable flavor	The delicate fragrance and pungent taste of vegetables.	Strong vegetable fragrance (5–9). Vegetable taste is low and it has a pungent spiciness (1–5).
Seafood flavor	Rich or light seafood flavor and fishy smell.	Has a good seafood flavor (6–9). Seafood taste is low and it is fishy (1–5).
Likeability	_____	Prefer (7–9), average (4–6), and dislike (1–3).

**Table 3:** Basic physicochemical parameters of different soy sauce.

Samples	L*	a*	b*	pH	Total Acid (Lactic Acid, g/100 mL)	TSS (°Brix)	Sodium Chloride (g/100 mL)	Non-Salt Soluble Solid (g/100 mL)	Amino Acid Nitrogen (g/100 mL)
S	34.29 ± 0.88 ^a^	−0.14 ± 0.07 ^ab^	−0.24 ± 0.06 ^e^	5.10 ± 0.03 ^e^	1.51 ± 0.02 ^a^	39.50 ± 0.05 ^a^	18.33 ± 0.55 ^a^	19.17 ± 0.20 ^a^	0.79 ± 0.04 ^a^
W1	24.39 ± 1.23 ^bc^	−0.28 ± 0.2 ^abc^	0.80 ± 0.16 ^cd^	5.11 ± 0.02 ^de^	0.92 ± 0.02 ^c^	27.88 ± 0.54 ^d^	12.87 ± 0.96 ^b^	11.43 ± 0.45 ^d^	0.49 ± 0.02 ^d^
CE1	25.33 ± 1.04 ^b^	−0.35 ± 0.11 ^bc^	0.72 ± 0.26 ^d^	5.12 ± 0.01 ^d^	1.04 ± 0.05 ^b^	28.25 ± 1.03 ^cd^	13.65 ± 0.55 ^b^	11.63 ± 0.13 ^cd^	0.59 ± 0.03 ^b^
CA1	25.44 ± 1.38 ^b^	−0.29 ± 0.28 ^abc^	1.10 ± 0.36 ^bc^	5.11 ± 0.01 ^de^	1.06 ± 0.02 ^b^	29.38 ± 0.96 ^bc^	13.26 ± 0.55 ^b^	12.32 ± 0.33 ^bc^	0.56 ± 0.01 ^c^
ON1	24.43 ± 1.56 ^bc^	−0.26 ± 0.12 ^ab^	1.00 ± 0.26 ^bcd^	5.11 ± 0.01 ^de^	1.01 ± 0.02 ^bc^	29.75 ± 0.25 ^b^	13.26 ± 0.55 ^b^	12.64 ± 0.25 ^b^	0.54 ± 0.01 ^c^
W2	25.69 ± 1.56 ^b^	−0.49 ± 0.28 ^c^	0.67 ± 0.17 ^d^	5.16 ± 0.01 ^c^	0.52 ± 0.02 ^d^	17.75 ± 0.83 ^f^	8.78 ± 0.59 ^c^	3.08 ± 0.05 ^g^	0.25 ± 0.01 ^e^
CE2	22.81 ± 1.99 ^c^	−0.27 ± 0.09 ^abc^	1.61 ± 0.46 ^a^	5.22 ± 0.01 ^b^	0.54 ± 0.05 ^d^	20.25 ± 0.25 ^e^	8.97 ± 0.55 ^c^	5.81 ± 0.02 ^f^	0.25 ± 0.01 ^e^
CA2	25.72 ± 1.34 ^b^	−0.17 ± 0.13 ^ab^	1.90 ± 0.25 ^a^	5.24 ± 0.03 ^a^	0.56 ± 0.02 ^d^	20.75 ± 0.56 ^e^	8.97 ± 0.55 ^c^	6.51 ± 0.13 ^ef^	0.24 ± 0.02 ^ef^
ON2	26.23 ± 2.1 ^b^	−0.10 ± 0.14 ^a^	1.28 ± 0.12 ^b^	5.22 ± 0.02 ^b^	0.50 ± 0.05 ^d^	20.75 ± 0.56 ^e^	8.58 ± 0.55 ^c^	6.65 ± 0.13 ^e^	0.22 ± 0.01 ^f^

Notes: Means with different letters in the same column are statistically significant at *p* < 0.05.

**Table 4:** Free amino acid content of soy sauce with different formulations.

Taste	FAAs (g/100 mL)	S	W1	CE1	CA1	ON1	W2	CE2	CA2	ON2
Umami	Asp	0.50 ± 0.03 ^a^	0.36 ± 0.01 ^cd^	0.40 ± 0.01 ^b^	0.38 ± 0.01 ^bc^	0.34 ± 0.00 ^d^	0.17 ± 0.01 ^e^	0.18 ± 0.01 ^e^	0.18 ± 0.00 ^e^	0.18 ± 0.01 ^e^
Umami	Glu	4.34 ± 0.03 ^a^	3.10 ± 0.09 ^c^	3.46 ± 0.09 ^b^	3.40 ± 0.07 ^b^	3.02 ± 0.03 ^c^	1.52 ± 0.12 ^d^	1.60 ± 0.05 ^d^	1.57 ± 0.02 ^d^	1.57 ± 0.04 ^d^
	Relative	61.58%	61.57%	61.66%	61.97%	61.76%	61.23%	59.73%	59.52%	59.12%
Sweet	Ser	0.10 ± 0.01 ^a^	0.06 ± 0.00 ^c^	0.07 ± 0.00 ^b^	0.09 ± 0.00 ^a^	0.07 ± 0.00 ^b^	0.04 ± 0.01 ^d^	0.04 ± 0.00 ^d^	0.04 ± 0.00 ^d^	0.04 ± 0.00 ^d^
Sweet/Bitter	Lys	0.29 ± 0.02 ^a^	0.20 ± 0.01 ^c^	0.22 ± 0.00 ^b^	0.23 ± 0.01 ^b^	0.20 ± 0.00 ^c^	0.10 ± 0.01 ^d^	0.11 ± 0.00 ^d^	0.11 ± 0.00 ^d^	0.11 ± 0.00 ^d^
Sweet/Bitter	Pro	0.32 ± 0.03 ^a^	0.24 ± 0.01 ^b^	0.25 ± 0.00 ^b^	0.15 ± 0.03 ^c^	0.14 ± 0.01 ^c^	0.08 ± 0.02 ^d^	0.09 ± 0.01 ^d^	0.10 ± 0.01 ^d^	0.10 ± 0.00 ^d^
Sweet	Gly	0.20 ± 0.01 ^a^	0.14 ± 0.00 ^c^	0.16 ± 0.00 ^b^	0.17 ± 0.00 ^b^	0.15 ± 0.00 ^c^	0.08 ± 0.00 ^e^	0.10 ± 0.00 ^d^	0.10 ± 0.00 ^d^	0.10 ± 0.01 ^d^
Sweet	Thr	0.22 ± 0.01 ^a^	0.16 ± 0.00 ^c^	0.18 ± 0.00 ^b^	0.17 ± 0.00 ^b^	0.15 ± 0.00 ^c^	0.08 ± 0.01 ^d^	0.09 ± 0.00 ^d^	0.09 ± 0.00 ^d^	0.08 ± 0.01 ^d^
Sweet	Ala	0.28 ± 0.02 ^a^	0.20 ± 0.01 ^c^	0.23 ± 0.01 ^b^	0.23 ± 0.00 ^b^	0.20 ± 0.00 ^c^	0.10 ± 0.01 ^d^	0.11 ± 0.00 ^d^	0.11 ± 0.00 ^d^	0.11 ± 0.00 ^d^
	Relative	17.94%	17.79%	17.73%	17.05%	16.73%	17.39%	18.12%	18.71%	18.24%
Bitter	Tyr	0.08 ± 0.01 ^a^	0.06 ± 0.00 ^b^	0.07 ± 0.00 ^b^	0.07 ± 0.00 ^b^	0.06 ± 0.00 ^b^	0.03 ± 0.00 ^c^	0.04 ± 0.00 ^c^	0.04 ± 0.00 ^c^	0.04 ± 0.00 ^c^
Bitter	Val	0.31 ± 0.02 ^a^	0.22 ± 0.01 ^c^	0.25 ± 0.01 ^b^	0.25 ± 0.00 ^b^	0.22 ± 0.00 ^c^	0.11 ± 0.01 ^d^	0.12 ± 0.00 ^d^	0.12 ± 0.00 ^d^	0.12 ± 0.01 ^d^
Bitter	Met	0.07 ± 0.01 ^a^	0.05 ± 0.00 ^c^	0.06 ± 0.00 ^b^	0.06 ± 0.00 ^b^	0.05 ± 0.00 ^c^	0.02 ± 0.00 ^e^	0.03 ± 0.00 ^d^	0.03 ± 0.00 ^d^	0.03 ± 0.00 ^d^
Bitter	Phe	0.24 ± 0.02 ^a^	0.17 ± 0.01 ^c^	0.19 ± 0.00 ^b^	0.19 ± 0.01 ^bc^	0.17 ± 0.00 ^c^	0.09 ± 0.01 ^d^	0.09 ± 0.00 ^d^	0.09 ± 0.00 ^d^	0.09 ± 0.00 ^d^
Bitter	Ile	0.27 ± 0.02 ^a^	0.19 ± 0.01 ^c^	0.21 ± 0.00 ^b^	0.21 ± 0.00 ^b^	0.19 ± 0.00 ^c^	0.10 ± 0.01 ^d^	0.10 ± 0.00 ^d^	0.10 ± 0.00 ^d^	0.10 ± 0.00 ^d^
Bitter	Leu	0.42 ± 0.03 ^a^	0.30 ± 0.01 ^c^	0.33 ± 0.01 ^b^	0.33 ± 0.01 ^b^	0.29 ± 0.00 ^c^	0.15 ± 0.01 ^d^	0.16 ± 0.01 ^d^	0.16 ± 0.00 ^d^	0.16 ± 0.01 ^d^
Bitter	His	0.10 ± 0.01 ^a^	0.07 ± 0.00 ^bc^	0.08 ± 0.00 ^b^	0.07 ± 0.00 ^bc^	0.06 ± 0.00 ^c^	0.04 ± 0.00 ^d^	0.04 ± 0.00 ^d^	0.04 ± 0.01 ^d^	0.04 ± 0.00 ^d^
Bitter	Arg	0.12 ± 0.01 ^a^	0.09 ± 0.00 ^c^	0.11 ± 0.00 ^b^	0.11 ± 0.00 ^b^	0.11 ± 0.00 ^b^	0.05 ± 0.00 ^f^	0.08 ± 0.00 ^de^	0.08 ± 0.00 ^e^	0.09 ± 0.00 ^cd^
	Relative	20.48%	20.46%	20.77%	21.15%	21.14%	21.38%	22.15%	22.45%	22.64%
Tasteless	Cys	0.01 ± 0.01 ^a^	0.01 ± 0.00 ^a^	0.01 ± 0.00 ^ab^	0.01 ± 0.00 ^ab^	0.01 ± 0.00 ^ab^	0.01 ± 0.01 ^ab^	0.00 ± 0.00 ^b^	0.00 ± 0.00 ^b^	0.01 ± 0.00 ^ab^
	Relative	0.13%	0.18%	0.16%	0.16%	0.18%	0.36%	0%	0%	0.34%
	Total Amino Acids	7.86 ± 0.55 ^a^	5.62 ± 0.17 ^cd^	6.26 ± 0.14 ^b^	6.10 ± 0.07 ^bc^	5.44 ± 0.04 ^d^	2.76 ± 0.22 ^e^	2.98 ± 0.05 ^e^	2.94 ± 0.03 ^e^	2.96 ± 0.10 ^e^

Notes: Means with different letters in the same column are statistically significant at *p* < 0.05.

## Data Availability

Data are available on request to the authors.
